# Reproductive physiological impacts of high ambient temperature on animals: the impaired testicular function and compromised sperm quality in C57BL/6 mice

**DOI:** 10.1186/s40659-025-00662-x

**Published:** 2025-12-23

**Authors:** Yun Ren, Kaixuan Zhang, Mengjiao Zhang, Yingying Xia, Yifeng Zhang, Jiqi Lu

**Affiliations:** 1 School of Life Sciences, Zhengzhou University, 100 Kexue Road, Zhengzhou, 450001 Henan China; 2 National Supercomputing Center in Zhengzhou, Zhengzhou University, 100 Kexue Road, Zhengzhou, 450001 Henan China

**Keywords:** High ambient temperature, Testicular injury, Sperm defects, Apoptosis, Oxidative stress

## Abstract

**Background:**

Global warming poses one of the most formidable challenges confronting the contemporary world, exerting extensive and profound impacts on both natural ecosystems and human society. Nevertheless, the detrimental effects of elevated temperatures on reproductive system in animals and humans remains elusive.

**Methods:**

In this study, C57BL/6 mice were exposed to high ambient temperatures (HAT). Subsequently, the testes and sperm parameters of the mice were evaluated following the exposure.

**Results:**

The HAT mice had significantly lower weight, food and water intake than those in the control group. The weight of testis and epididymis was significantly lower in the HAT group. The HAT group showed significant reductions in seminiferous tubule area, spermatogenic epithelium area, and seminiferous epithelium thickness. The HAT group exhibited significantly reduced sperm density, viability, and plasma membrane integrity rate. The HAT group exhibited a significantly elevated rate of sperm malformation. Moreover, the expression levels of Dnah8 and Dnah17, two genes associated with Multiple Morphological Abnormalities of the Flagella (MMAF), were markedly reduced in the HAT group. The proportion of TUNEL-positive cells significantly increased in the HAT group. Furthermore, there was a significant upregulation in mRNA expression levels of apoptotic genes Caspase-3 and Bax in the HAT group. Conversely, there was a notable decrease in mRNA expression level of anti-apoptotic gene Bcl-2. The concentration of MDA in the HAT group was significantly higher, while SOD activity and T-AOC were significantly lower.

**Conclusions:**

Therefore, it can be inferred that elevated air temperature leads to multi-layered damage to animal reproduction. This suggests that extreme high temperatures resulting from climate warming will have a detrimental impact on animal reproduction. This study provides support for elucidating the mechanism underlying the reproductive effects of high temperature.

**Supplementary Information:**

The online version contains supplementary material available at 10.1186/s40659-025-00662-x.

## Introduction

Over the course of the last century, global surface temperatures have risen by approximately 1 °C, with projections indicating a potential increase exceeding 1.5–2 °C by the end of the 21st century [1]. This warming trend is contributing to more frequent and intense heatwaves [2], creating formidable challenges for species worldwide [3–6]. Among these challenges, the impact of rising temperatures on reproductive health has become increasingly apparent, as reproduction—a critical process for the survival and sustainability of species—is particularly vulnerable to thermal stress. Evidence suggests that elevated temperatures can negatively affect reproductive systems across a wide range of species [7–10]. Such impacts on reproduction not only threaten animal populations but also have significant implications for biodiversity.

In parallel, infertility has emerged as a prevalent condition affecting couples in their reproductive phase [11, 12]. Epidemiological surveys report an incidence rate of infertility from 8 to 12%, with approximately 50% of cases attributed to male factors [13]. Extended exposure to high-temperature environments, whether due to climate change or specific occupational hazards, has been shown to have a detrimental effect on male reproductive function [14]. For instance, steel mill workers engaged in high-temperature occupational settings exhibit a decline in sperm quality compared to their counterparts in cooler working conditions [15]. Similarly, individuals in the bread baking industry, facing prolonged exposure to heightened temperatures, may experience an increased risk of infertility [16]. Furthermore, high temperatures have been associated with adverse pregnancy outcomes, underscoring the broader reproductive implications of heat exposure across different contexts [17].

The process of spermatogenesis is highly dependent on appropriate temperature regulation [18], with the optimal temperature being slightly lower than core body temperature. The testis maintains a temperature 2–7 ℃ lower than core body temperature through a specialized heat exchange mechanism, which is crucial for normal production of sperm [19]. A transient increase of merely 2 ℃ can elicit a substantial 25-fold increase in double-strand DNA breaks in the germline cells of Caenorhabditis elegans during the meiotic prophase [20]. Furthermore, exposure to temperatures ranging between 37–38 ℃ disrupts the first stage of meiotic division in mice, leading to apoptotic cell death in spermatocytes, disruption of spermatogenesis, and consequent infertility [21]. In the context of rats, heat stress disrupts the spermatogenesis process by impairing the specific calcium ion channels that are distinctive to sperm [22].

Despite previous findings, comprehensive studies that systematically examine the multifaceted impacts of elevated temperatures on testicular function in mammals remain limited. We therefore hypothesized that heatwave-like environmental exposure would adversely affect male reproductive function by disrupting spermatogenesis and inducing structural and molecular alterations in the testes. To test this hypothesis, the present study employed a controlled heatwave-simulation model in C57BL/6 mice and assessed the effects of elevated ambient temperature on sperm quality, testicular morphology, antioxidant capacity, and cellular apoptosis. These experiments are essential for addressing the broader implications of climate change on reproductive health.

## Materials and methods

### Animals and treatment

The experimental animals consisted of 40 healthy male C57BL/6 mice (SPF grade) obtained from Henan Experimental Animal Centre, Zhengzhou, China) at the age of 8 weeks. Upon arrival, the mice were group-housed in standard individually ventilated cages with corncob bedding and sterilized nesting paper provided. Before the beginning of heat exposure, all animals were transferred to a constant temperature and humidity climatic chamber and allowed to acclimate for 7 days.

After acclimation, these mice were randomly assigned into two groups of 20 mice each, with an average weight of 20.60  ±  0.30 g. The animals were housed in a controlled environment with a 50% humidity, maintained on a 12-h light and dark cycle, with ad libitum access to free water and food. The mice in both groups were fed a basal diet (produced by Henan Experimental Animal Centre, Zhengzhou, China) for a total of 5 weeks.

For heat treatment, mice were single-housed (one mouse per cage), and the temperature within the heat-exposure chamber was elevated to 38 ℃ for three hours daily from 11:00 to 14:00, while the control chamber remained at 25 ℃. Chamber temperature was continuously monitored and maintained within ± 0.5 °C of the setpoint throughout the exposure period to ensure stable and biologically relevant heat stress. The animals were closely monitored and scarified at 35 days post treatment. The research procedures underwent review and approval by Zhengzhou University’s animal care and use committee (Permit Protocol Number ZZUIRB2021-125).

### Histological morphological analysis of the testis

In order to conduct morphological analysis, the left testis was fixed in 4% paraformaldehyde for 24 h. The fixed tissue was subsequently dehydrated using a series of graded alcohol concentrations. Subsequently, the testis was made transparent using xylene and embedded in paraffin and Hematoxylin and eosin (H&E) staining was performed. Transverse sections were cut at a thickness of 5 μm using a Leica HistoCore BIOCUT rotary microtome (Leica Microsystems, Germany). The paraffin sections were dewaxed twice in xylene, dehydrated with a gradient of ethanol, and then stained with hematoxylin. The stained sections were differentiated with 1% hydrochloric acid in ethanol and counterstained with eosin. Afterward, the sections were washed in ethanol solutions with different concentrations for 5 min each. The sections were then made transparent in xylene and mounted with neutral gum.

The morphology of testicular tissue was observed using an optical microscope (BX53, Olympus, Japan). Five mice were randomly selected from each group, and four sections were chosen from each mouse. Representative seminiferous tubules were photographed using ImageJ software (version 1.53t, National Institutes of Health, USA) to measure their diameter (µm) and count the number of germ cells in 20 randomly selected fields of view. This methodology was employed to facilitate morphological examination of the tissue and to ensure optimal preservation of the cellular structure.

### Testicular apoptosis analysis

TUNEL staining was performed on testicular cells using a commercial apoptosis detection kit (A112, Vazyme Biotech Co., Ltd, Nanjing, China) according to the manufacturer’s instructions. Testicular tissues were fixed and prepared as described above. Testicular sections were dehydrated in an ethanol gradient (100 − 70%), incubated in 1% Triton X-100 at room temperature for 30 min, and then incubated in a 3% H_2_O_2_-methanol solution at room temperature for 10 min. Proteinase K was added to the sections and incubated for 15 min. The sections were then incubated with TdT enzyme at 37 ℃ for 1 h, washed three times with PBS (each time for 5 min), and counterstained with DAPI to stain the nuclei. Five randomly selected fields of view were observed under a laser confocal microscope (BX53, Olympus, Japan), and the TUNEL-positive rate of cells within seminiferous tubules was calculated.

### Sperm quality and motility assessment

The epididymides of each mouse were collected and the left epididymal tail was minced in 1 mL of physiological saline. The resulting tissue was incubated at 37 °C water bath for 30 min to release the sperm. Sperm counting was performed using a preheated hemocytometer, and motility assessment was conducted under an optical microscope. Modified Papanicolaou staining was used to observe and calculate the sperm abnormality rate. Sperm tail membrane integrity was assessed through the sperm hypo-osmotic swelling test. All procedures were performed by a trained experimenter to ensure consistency.

### Evaluation of oxidative stress

At the conclusion of the 5-week experimental period for each group of mice, the mice were subjected to a 12-hour fasting period without restricting water access. Subsequently, they were intraperitoneally injected with an appropriate dose of 3% pentobarbital sodium solution based on their body weight. Following complete anesthesia of the mice, blood samples were collected via retro-orbital puncture. The collected blood was then transferred into 1.5 mL centrifuge tubes and allowed to stand at room temperature for 0.5 h. Subsequently, the samples were centrifuged at 5000 rpm for 20 min, and the resulting supernatant was aliquoted into 200 µL centrifuge tubes, labeled, and stored in a -80 °C freezer until needed for measure of SOD activity, malondialdehyde (MDA) and total antioxidant capacity (T-AOC).

SOD activity was assessed using a commercial assay kit (A001-3-2, Nanjing Jiancheng Bioengineering Institute, Nanjing, China). The concentration of malondialdehyde (MDA), a product of lipid peroxidation, was determined using an MDA assay kit (A0003-1-2, Nanjing Jiancheng Bioengineering Institute, Nanjing, China). The levels of T-AOC in serum were measured following the instructions provided by the assay kit (A015-1-1, Nanjing Jiancheng Bioengineering Institute, Nanjing, China). A single Trolox standard curve was generated and used to calculate T-AOC for all samples; results are reported as **mM Trolox equivalents (mM Trolox equiv.)**. Absorbance for all three assays was measured using a K3 TOUCH microplate reader (Thermo Fisher Scientific, USA).

### RNA isolation and real-time PCR

In accordance with the manufacturer’s guidelines, the extraction of total RNA was carried out using the Total RNA Isolation Kit (Bei-Bei BIOTECH, Zhengzhou, China). Subsequent cDNA synthesis was achieved utilizing the HiScript Ⅲ All-in-one SuperMix Perfect for qPCR (R333-01, Nanjing Vazyme Biotech Co., Ltd, Nanjing, China).

Real-time quantitative PCR (RT-qPCR) was performed on a LightCycler^®^ 480 Real-Time PCR system (Roche, Switzerland) using SYBR Green Master Mix (Vazyme, China). The amplification program was set as follows: 95 °C for 10 min, followed by 40 cycles of 95 °C for 10 s, 56 °C for 30 s and 72 °C for 36 s. A melting curve analysis was conducted at the end of amplification to confirm product specificity. GADPH was used as the internal reference gene, and relative expression levels were calculated using the 2^ − ΔΔCt method. Detailed primer sequences are provided in Supplementary Table S1.

### Blinding and data collection

Data collection and analysis were conducted under partially blinded conditions. For subjective evaluations, including sperm count, motility, abnormality rate, membrane integrity, and histological analyses (H&E and TUNEL staining), the investigators were blinded to group allocation during data acquisition and scoring. For objective measurements, such as body and organ weights, biochemical assays (SOD, T-AOC, and MDA), and gene expression analysis, blinding was not applied, as these endpoints were quantified using automated or spectrophotometric readouts. All procedures were performed by trained experimenters following standardized protocols to ensure consistency and minimize bias.

### Data analysis

All data were analyzed using GraphPad Prism 9.5.1 (GraphPad Software, USA). Quantitative data were tested for normality before statistical analysis. Comparisons between two groups were performed using unpaired two-tailed Student’s t-tests. Data are presented as mean ± standard deviation (SD), and statistical significance was defined as *P* < 0.05. Figures and graphs were generated using GraphPad Prism.

## Results

### Effect of HAT on intake, body weight and organ indices of mice

**Fig. 1:**
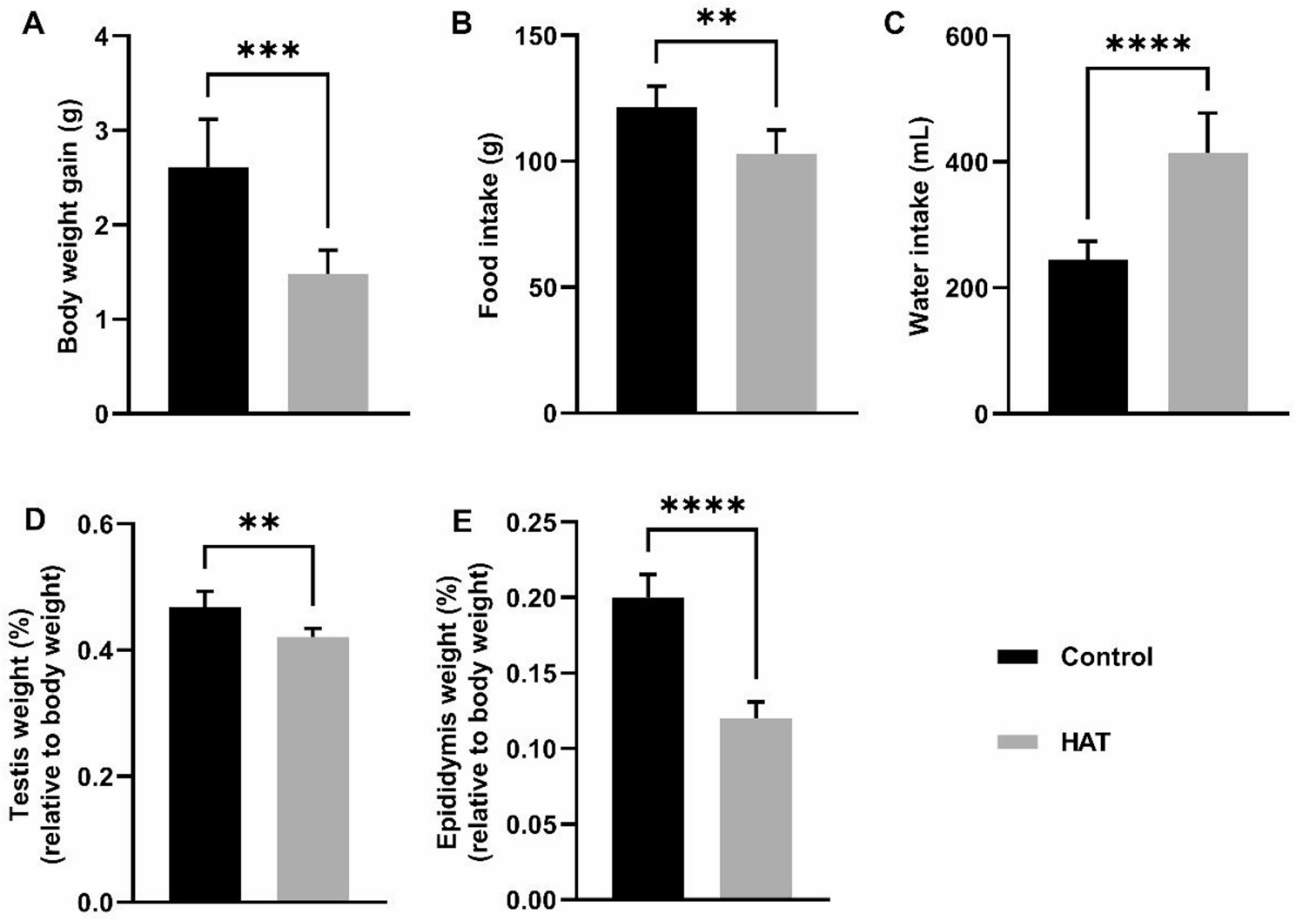
Changes in mice body weight, consumption and reproductive organ weights in mice after HAT exposure. **A** Body weight gain in control and HAT-treated mice post-experiment, (Con *n* =  7, HAT *n*  = 7). **B**,** C** Cumulative food intake (**B**) and water consumption (**C**), (Con *n* =  7, HAT *n* = 7). **D**,** E** Testicular (**D**) and epididymal (**E**) weights relative to body weight, Sample sizes: Testicular relative weights (Con *n* = 6, HAT *n* = 7); epididymal relative weights (Con *n* = 7, HAT *n* =  7). Data in panels** A**–**E** are presented as means with standard deviations. Statistical analysis was performed using unpaired two-tailed *t*-test. The asterisks (*, **, ***, ****) denote statistical significance levels of *P* <  0.05, *P* <  0.01, *P* <  0.001, and *P* < 0.0001, respectively. Student’s* t*-test for comparison with the control. Throughout the figures in this article, “Control” represents the control group, and “HAT” represents the high ambient temperature exposure group.

In our exploration of the physiological impact of HAT (High ambient temperature) on mice, we analyzed changes in parameters such as body weight, food intake, and water consumption. Initially, both groups of mice exhibited no discernible differences in body weight. However, the HAT group demonstrated a significant reduction in the rate of body weight gain compared to the control group (Fig. 1A). Additionally, there was a marked reduction in food intake within the high-temperature group (Fig. 1B). In contrast, the HAT group exhibited a noteworthy increase in water consumption relative to the control group (Fig. 1C). The HAT exposure also led to reductions in testicular and epididymal weight, indicating detrimental effects on male reproductive organs (Fig. 1D, E). In addition, the weights of spleen, lung and kidney were notably reduced, while no significant changes were observed in the heart and liver (Fig. S1). These observations collectively underscore the profound physiological alterations induced by elevated temperature in our experimental mouse cohort.

### HAT damages testicular structural integrity

**Fig. 2:**
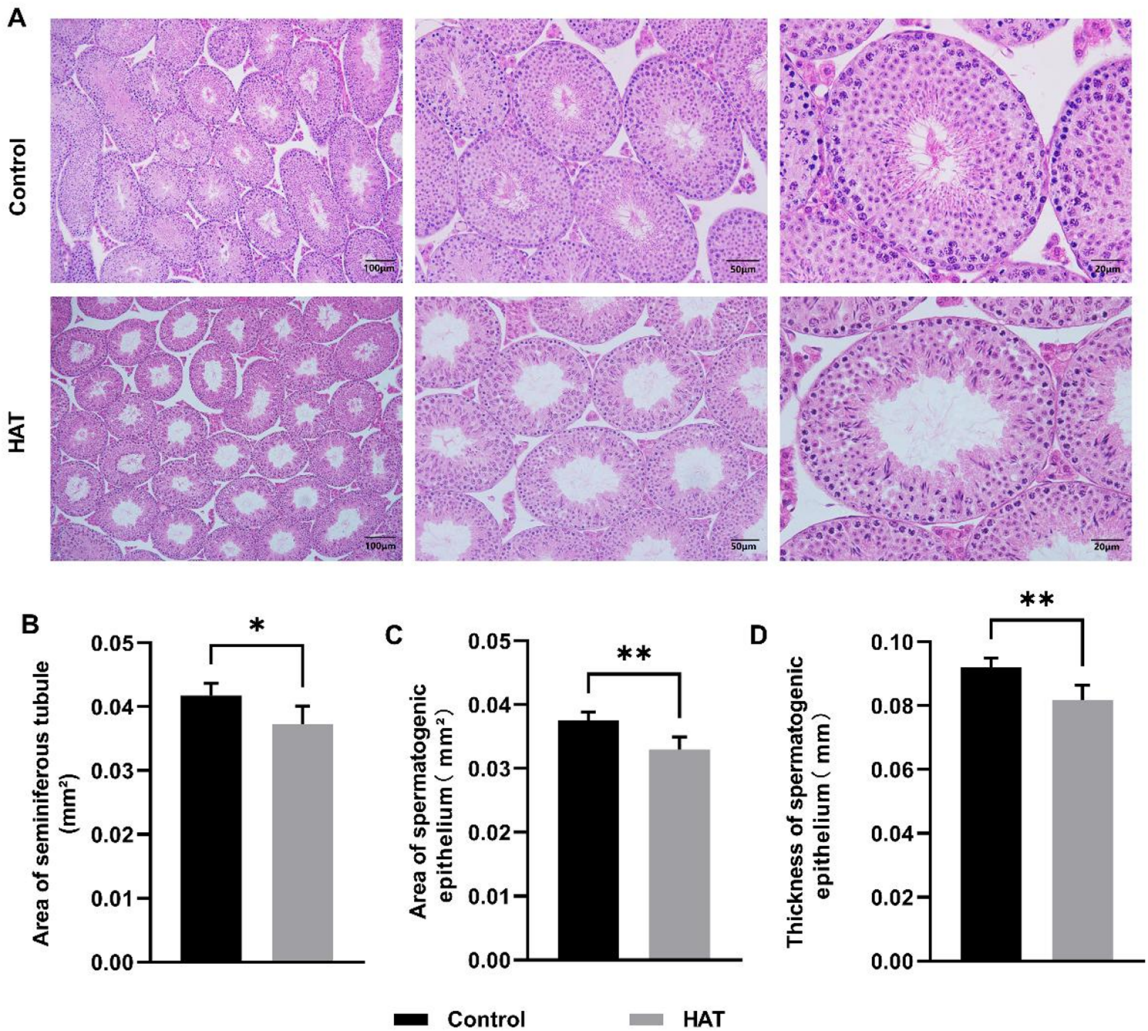
Testicular injury induced by HAT exposure in mice, (Con *n* = 5, HAT *n* = 5). **A** Morphology assessments of testicular tissue from control and HAT groups, as illustrated by H&E staining. Scale bars: 100 μm (left panels), 50 μm (middle panels) and 20 μm (right panels).** B**,** C**,** D** Quantitative evaluation of the seminiferous tubules area (**B**), spermatogenic epithelium area (**C**) and seminiferous epithelium thickness (**D**). Statistical analysis was performed using unpaired two-tailed *t*-test.

Histological examination revealed distinct differences between the control and HAT groups in testicular sections (Fig. 2A). In the control group, the seminiferous tubules exhibited organized arrangement of germ cells, with a substantial presence of spermatozoa within the lumen. The Sertoli cells displayed irregular triangular or stellate shapes with relatively large cytoplasm, and their nuclei were over in shape. The spermatogenic cells were close to the basement membrane of the seminiferous tubules, located between the Sertoli cells, and exhibited deep nuclear staining. In contrast, in the HAT group, the intertubular spaces of the seminiferous tubules increased, accompanied by the appearance of vacuoles. The number of germ cells was significantly reduced, with some cells detached and entering the lumen. The cellular arrangement in the testes became more loosely packed, and the connections between Sertoli cells were disrupted. In comparison to the control group, the HAT group exhibited a reduction in the area of seminiferous tubules, accompanied by a decrease in both the area and thickness of the seminiferous epithelium (Fig. 2B, C, D).

### HAT decreases sperm quality in mice

**Fig. 3:**
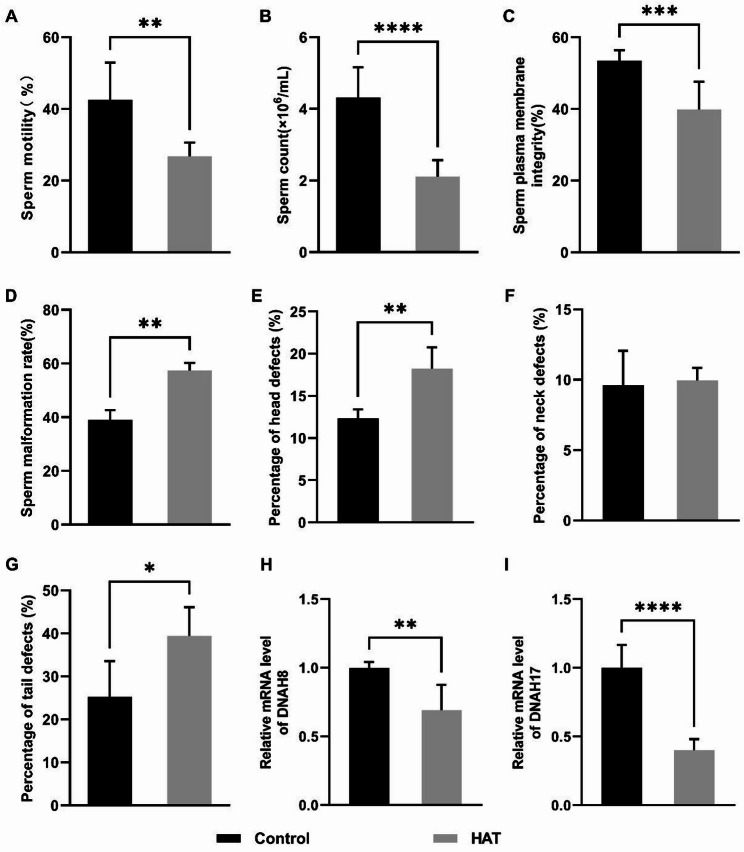
Impact of HAT exposure on mouse sperm quality. **A** Sperm count in the Control and HAT groups, (CON *n* = 6, HAT *n* = 10). **B** Sperm motility, (CON *n* = 7, HAT *n* = 6). **C** Sperm membrane integrity, (CON *n* = 9, HAT *n* = 10). **D** Sperm abnormality rate. The epididymis was carefully dissected and the sperm count per hamster testicle was determined, (Con *n* = 3, HAT *n* = 3). **E**,** F**,** G** The percentage of sperm head (**E**), neck (**F**), and tail G abnormality, (Con *n* = 4, HAT *n* = 4 ). **H**,** I** mRNA expression of DNAH8 (**H**) and DNAH17 (**I**), (*n* = 6). Statistical analysis was performed using unpaired two-tailed *t*-test.

Comparison between the control and the HAT groups revealed significant differences in both sperm quantity and quality. The HAT group showed a marked reduction in sperm count and an increase in sperm abnormality rates compared to the control group (Fig. 3B, D). Additionally, there was a notable decline in overall sperm motility and plasma membrane integrity in the HAT group (Fig. 3A, C).

Abnormality rates in various regions of sperm were subjected to statistical analysis, revealing a noteworthy increase in both head and tail abnormality rates within the HAT group (Fig. 3E, G). Conversely, the abnormality rate in the neck region showed no significant difference compared to the control group (Fig. 3F, Fig. S2).

Multiple Morphological Abnormalities of the Flagella (MMAF) represent a severe manifestation of asthenoteratozoospermia, primarily characterized by diverse abnormalities in the morphology of sperm tail flagella. These abnormalities include tailless, short-tailed, coiled, bent, and irregularly shaped tails, often accompanied by reduced sperm motility or a reduction in sperm count [23]. We assessed the mRNA expression levels of DNAH8 and DNAH17, two genes associated with MMAF, and observed a downregulation in the expression of both genes in the HAT group (Fig. 3H, I).

### HAT exposure induces testicular apoptosis

**Fig. 4:**
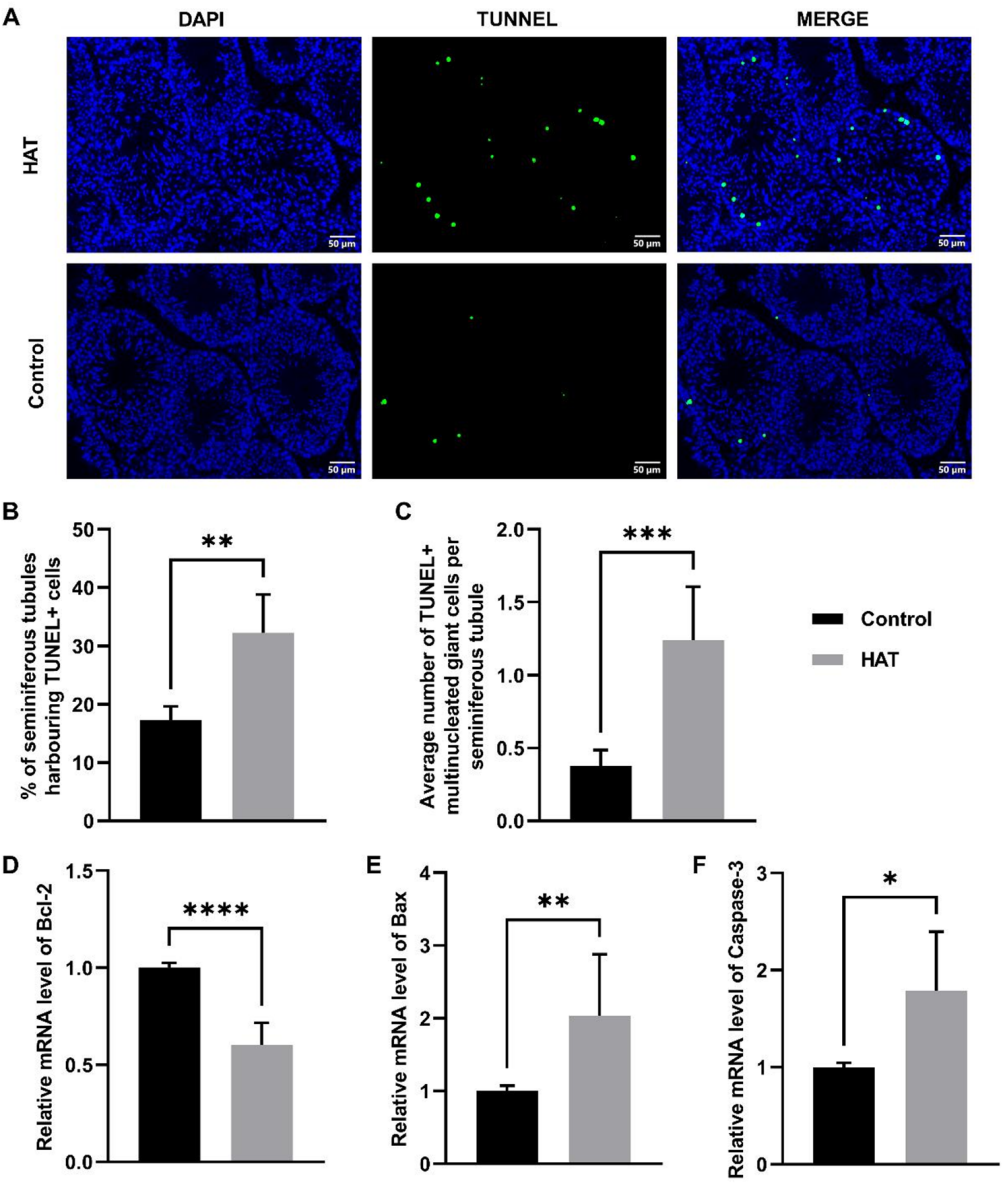
Apoptosis in testis tissue after HAT exposure to 25 ℃ or 38 ℃ air.** A** TUNEL staining analysis of testicular apoptosis in control and HAT group. Scale bar = 50 μm. DAPI staining results in blue fluorescence, indicating normal nuclei, while TUNEL staining produces green fluorescence, highlighting apoptotic cells. **B** The proportion of seminiferous tubules containing apoptotic cells in each group, (Con *n* = 4, HAT *n* = 4). **C** Average number of apoptotic multinucleated giant cells per seminiferous tubules, (Con *n* = 5, HAT *n* = 5). **D**–**F** mRNA expression level of apoptosis-related genes. Sample sizes: Bcl2 (Con *n* = 6, HAT *n* = 8); Bax (Con *n* = 9, HAT *n* = 6); Caspase-3 (Con *n* = 6, HAT *n* = 5). Statistical analysis was performed using unpaired two-tailed *t*-test.

To explore heat-induced germ cell apoptosis, TUNEL was performed on testicular sections to detect sperm DNA breakage levels, and it was found that the proportion of TUNNEL-positive cells increased in the HAT group (Fig. 4A–C, Fig. S3), indicating that HAT promotes apoptosis in testicular cells.

Furthermore, the expression of apoptosis-related genes was measured to validate the occurrence of apoptosis. Compared to the control group, the HAT group showed a significant increase in the mRNA expression levels of apoptotic genes Caspase-3 and Bax. Conversely, the mRNA expression level of anti-apoptotic gene Bcl-2 was notably decreased. These results suggest that heat stress induces a shift towards pro-apoptotic signaling.

### HAT exposure induces oxidative stress

**Fig. 5:**
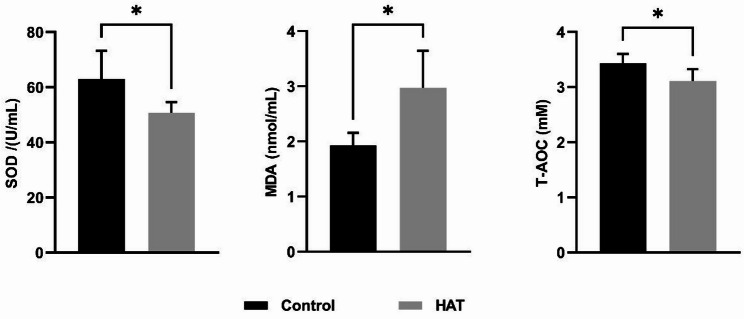
Effect of HAT treatment on levels of SOD, MDA and T-AOC in the plasma of mice. Sample sizes: SOD (Con *n* = 6, HAT *n* = 6); MDA (Con *n* = 4, HAT *n* = 4); T-AOC (Con *n* = 6, HAT *n* = 5). Statistical analysis was performed using unpaired two-tailed *t*-test.

Oxidative stress is a significant factor contributing to sperm dysfunction [24] and plays a crucial role in heat stress-induced cell apoptosis [25]. Superoxide dismutase (SOD) is an important antioxidant enzyme in organisms that scavenges superoxide anion radicals and protects cells from oxygen radical damage. Malondialdehyde (MDA) is a marker reflecting lipid peroxidation in oxidative stress. To assess the oxidative damage caused by high-temperature treatment, the levels of SOD and MDA were measured. Compared to the control group, the mice in HAT group displayed a significant elevation in the serum MDA content, indicative of a higher degree of lipid peroxidation within the organism. The SOD level significantly decreased after HAT treatment, reflecting the impaired oxidative capacity of the testes. Simultaneously, the overall antioxidant capacity (T-AOC) also decreased (Fig. 5).

## Discussion

Elevated temperatures have emerged as a significant concern in the context of global climate change, with potential impacts on various biological processes, including reproductive functions [7]. Climate-driven temperature increases can negatively affect normal reproductive processes, posing risks to both biodiversity and species survival [26, 27]. As such, understanding how rising temperatures affect reproductive health is crucial for comprehending the ecological consequences of climate change.

Our study utilized a climate-controlled chamber to expose mice to a temperature of 38 °C for 3 h daily over a period of 35 days. Comparable telemetry studies show that continuous ambient heating at ~ 37 °C can raise murine core temperature by ~ 2 °C [28], supporting the biological plausibility that environmental warming produces measurable internal thermal load. This moderate, sublethal heat exposure contrasts with acute scrotal heating above 40 °C, providing a sustained thermal load that allows assessment of long-term effects on spermatogenesis and testicular function.

In this context, our study sheds light on the detrimental effects of environmentally relevant heat exposure on male reproductive health. Prolonged exposure to elevated temperatures significantly impairs sperm quality and motility, induces oxidative stress, and contributes to testicular damage. These findings provide important insights into the reproductive risks posed by ongoing climate change, emphasizing the vulnerability of reproductive processes to thermal stress.

Following this heat exposure, we observed significant reductions in both testicular and epididymal weight, indicating clear detrimental effects on male reproductive organs. Histological assessments revealed further evidence of testicular damage, showing notable morphological alterations such as germ cell loss, disruption of cellular organization, and compromised integrity of the Sertoli cell network. Germ cells are particularly vulnerable to heat stress due to their active proliferation and reliance on Sertoli cells for structural and nutritional support [29].

Oxidative stress emerges as a critical factor contributing to testicular damage [30, 31]. High temperatures could impair mitochondrial electron transport chain function, causing electron leakage and excessive ROS formation. Additionally, the activity of certain antioxidant enzymes, such as superoxide dismutase and glutathione peroxidase, is inhibited under high-temperature conditions, reducing the cell’s ability to eliminate ROS and leading to oxidative stress [32].

The high content of polyunsaturated fatty acids in the sperm plasma membrane phospholipid renders it highly sensitive to ROS. Accumulated ROS can readily induce lipid peroxidation and impaired DNA integrity. ROS at physiological levels plays a role in many crucial biological processes in the testes, such as sperm capacitation, acrosome reaction, hyperactivation, and sperm-egg binding [33]. The excessive ROS generated as a result of high-temperature exposure have a significant impact on the regulation of reproductive cell apoptosis and DNA damage [34]. Consistent with these mechanisms, our findings show increased lipid peroxidation and reduced antioxidant capacity following heat exposure, supporting oxidative stress as a central pathway underlying the observed testicular damage.

ROS can attack DNA molecules, resulting in the formation of 8-hydroxy-2’-deoxyguanosine (8-OHdG), which may pair with A, T, and C, thereby causing genetic mutations. Gene mutations are crucial contributors to impaired sperm quality and male infertility [23, 35]. MMAF represent one of the most severe sperm tail defect phenotypes, characterized by missing, coiled, short tail, or irregular flagella, thereby affecting sperm motility and is a significant contributing factor to male infertility [36, 37]. Previous genetic studies have identified several Dynein Axonemal Heavy Chain (DNAH) family members as key regulators of axonemal structure. For instance, knockout of DNAH8 in mice leads to typical MMAF defects and infertility [38], while loss-of-function mutations in DNAH17 cause isolated male infertility with MMAF phenotypes in humans [39].

In our study, heat exposure increased the proportion of abnormal sperm tails, including coiled and bent morphology, and was accompanied by reduced expression of DNAH8 and DNAH17. These results collectively suggest that heat stress may contribute to MMAF-like phenotypes. This substantiates existing understanding regarding the susceptibility of spermatogenesis to thermal stress.

The present study demonstrates that elevated temperature significantly impairs male reproductive health, as evidenced by abnormal sperm morphology and reduced sperm quality, and is associated with MMAF-like phenotypes under prolonged exposure. Although the present study demonstrates that heat exposure is associated with increased oxidative stress and impaired sperm quality, the data do not allow us to establish a direct causal relationship between these processes. The specific signaling events linking heat-induced oxidative stress to the observed sperm defects have not yet been delineated. Future work should investigate heat-responsive pathways—such as p53-BAX-caspase apoptosis signaling—to determine whether their activation directly contributes to the morphological and functional abnormalities observed. Clarifying these mechanisms will be essential for establishing causality in heat-induced male reproductive impairment.

It should be noted that testicular changes were evaluated only after 35 days of exposure. Testicular responses to heat may vary over time and with different exposure intensities. Therefore, future studies involving multiple time points, varying heat doses, and recovery periods would be valuable to elucidate the dynamics, dose-response and reversibility of these effects. In addition, the study is limited by the relatively small sample sizes in certain assays and the lack of functional fertility assessments. Extrapolation of these findings to humans should be approached with caution due to physiological differences between species.

Future investigations should explore whether heat-induced reproductive impairments are reversible and identify potential strategies for fertility rescue, such as antioxidant supplementation, thermal protection approaches, or targeted modulation of apoptosis-related pathways. Long-term follow-up studies assessing sperm function, testicular architecture, and reproductive capacity would further clarify the persistence and biological significance of heat-related reproductive damage.

## Conclusion

Collectively, these findings underscore the imperative for comprehensive investigations into the intricate molecular and physiological mechanisms through which elevated temperature exert their influence on male reproductive function. Our results suggest a potential association between heat stress and abnormalities in sperm flagellar structure, offering new insights that may help bridge current knowledge gaps. These insights are pivotal for understanding and addressing the potential impacts of climate-driven temperature increases on both human and wildlife populations.

## Supplementary Information

Below is the link to the electronic supplementary material.

Supplementary Material 1

## Data Availability

The data for this study are available within the article, with supplementary data provided in the Supporting Information. Additionally, data may be obtained from the corresponding author upon reasonable request.

## Abbreviations

HAT: High ambient temperature
MMAF: Multiple morphological abnormalities of the flagella
SOD: Superoxide dismutase
MDA: Malondialdehyde
ROS: Reactive oxygen species
8-OHdG: 8-hydroxy-2’-deoxyguanosine
