# Supplementary material for: Reproductive physiological impacts of high ambient temperature on animals: the impaired testicular function and compromised sperm quality in C57BL/6 mice
Source: Biol Res. 2025 Dec 23;59:5. doi: 10.1186/s40659-025-00662-x (PMC12836821; doi:10.1186/s40659-025-00662-x)
Supplement: Supplementary file 1 — Supplementary Material 1 [file 40659_2025_662_MOESM1_ESM.docx]

Supplementary information

Figure S1 Organ index of Control and HAT groups.

Figure S2 Sperm morphology in HAT group.

Figure S3 Representative TUNEL staining of testicular sections in the HAT group.

Table S1 Primer sequences used for qPCR analysis.


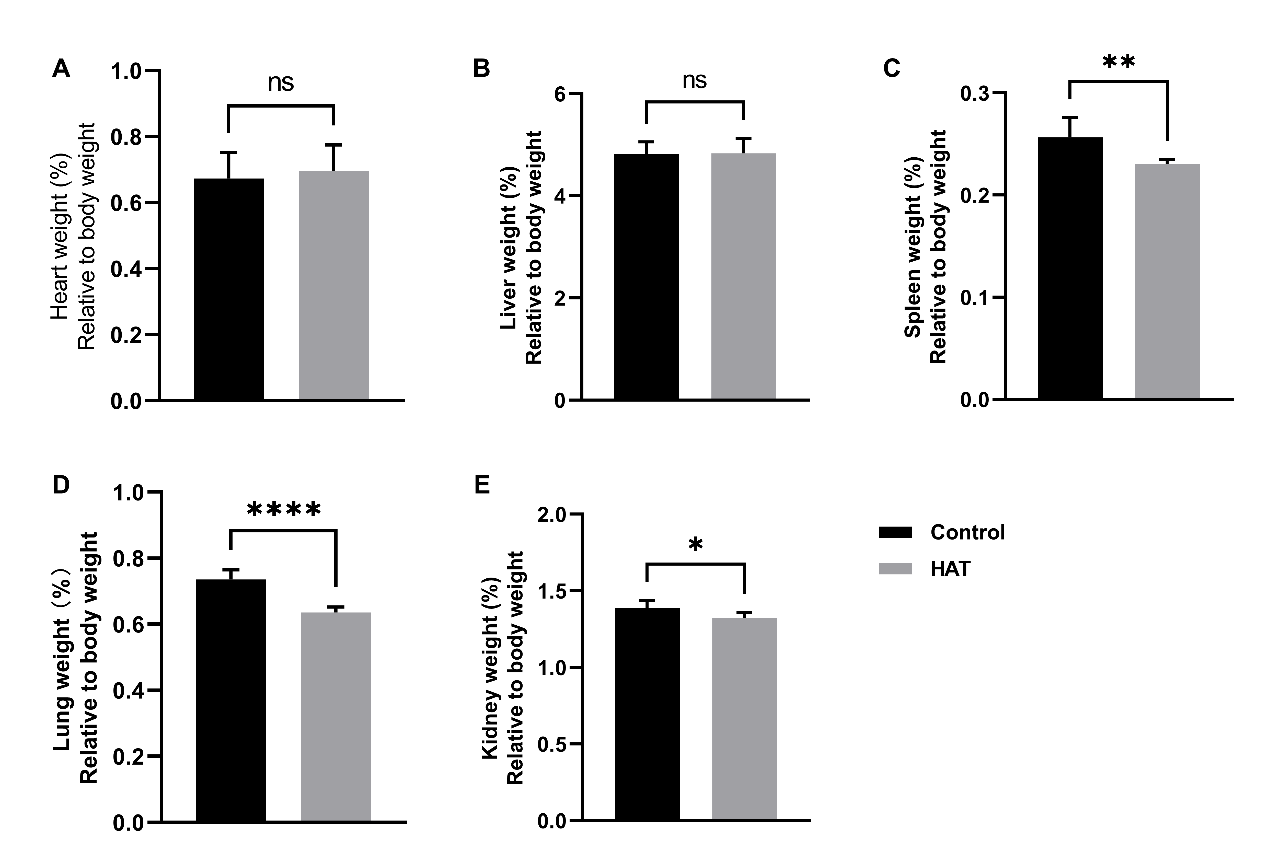


Figure S1 Organ index of Control and HAT groups.

(A) Heart weight, (B) Liver weight, (C) Spleen weight, (D) Lung weight and (E) Kidney weight of control and HAT-treated male mice. Tissue index was calculated by the following formula: Tissue index (%) = weight of tissue/weight of body *100%.


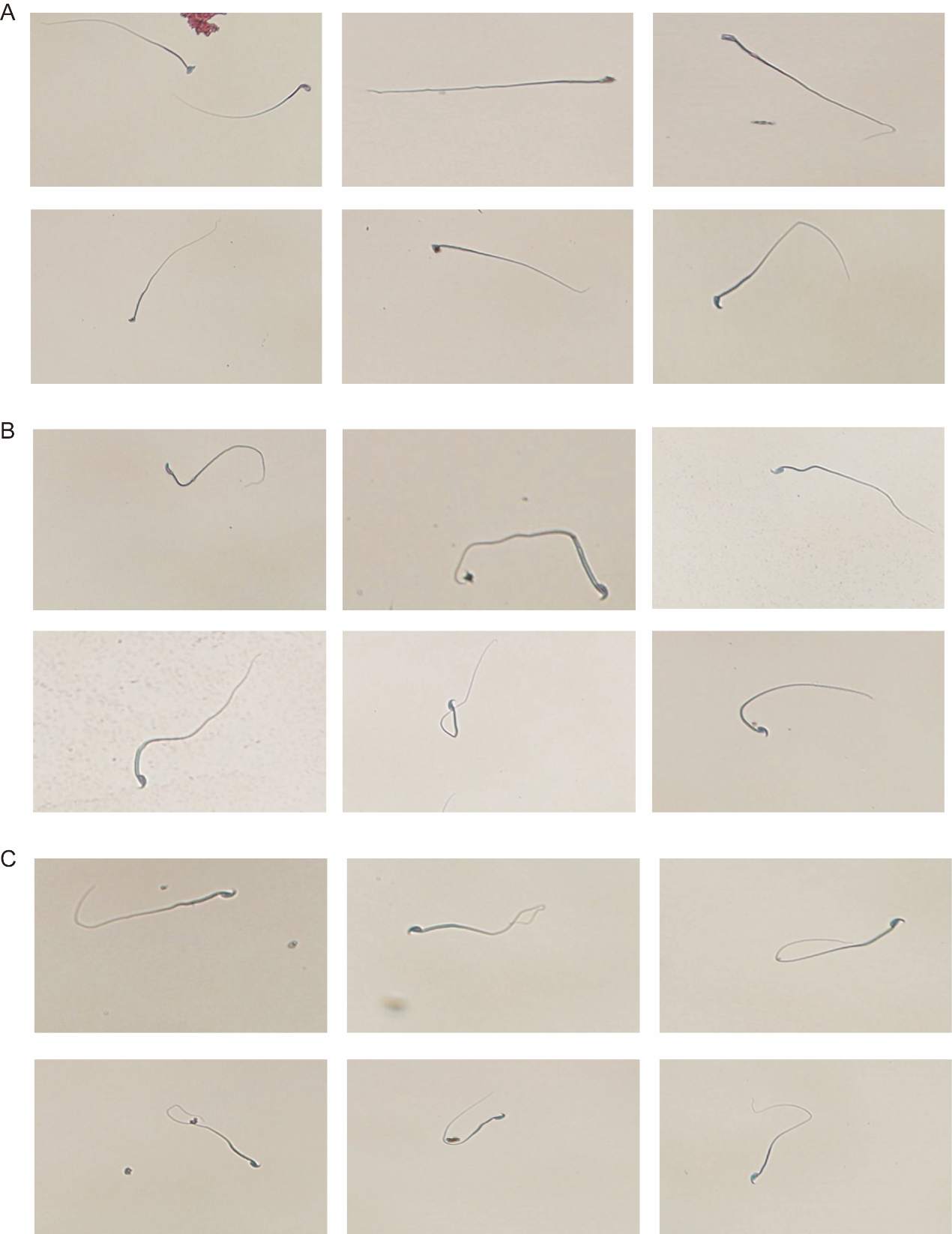


Figure S2 Representative images of sperm morphological abnormalities in the HAT group. Images are cropped from original microscopic fields for clear visualization of different abnormal phenotypes.

(A) Representative examples of head abnormalities.

(B) Representative examples of neck abnormalities.

(C) Representative examples of tail abnormalities.


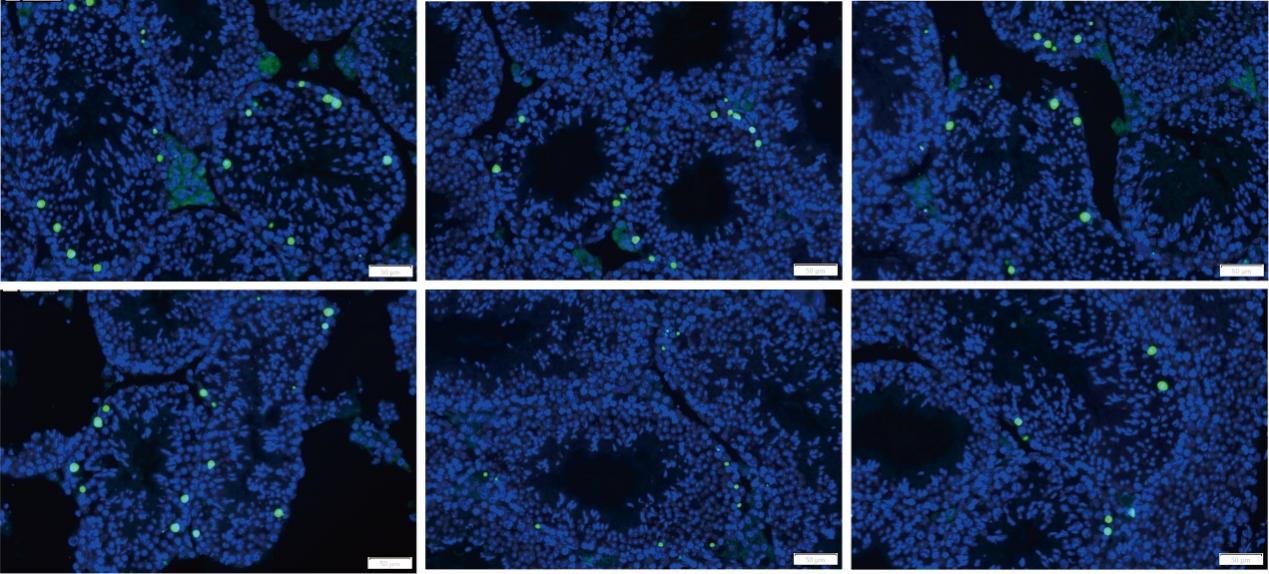


Figure S3 Representative TUNEL staining of testicular sections in the HAT group.

| Table S1 Primer sequences for Real-time Quantitative PCR | | |
| --- | --- | --- |
| Gene | Forward sequence（5‘-3’） | Reverse sequence（5‘-3’） |
| *BCL-2* | TGGGCCAGGGTGTGAAGGTCT | TGCAAAAAGTCCTCAGGCGAAC |
| *Caspase3* | TGTCATCTCGCTCTGGTACG | TCCCATAAATGACCCCTTCA |
| *BAX* | CACAGCGTGGTGGTACCTTA | TCTTCTGTACGGCGGTCTCT |
| *DNAH8* | ACCCCCTCCGAGAAGAG | TCAGTCACGGAAGTAGCAGGA |
| *DNAH17* | ATGATCACCGTGGAGAGTTCG | GACTGCGTGAGCGTGATGTA |
| *GAPDH* | ACCCAGAAGACTGTGGATGG | CACATTGGGGGTAGGAACAC |
